# Modulation of Phosphorylation of Tocopherol and Phosphatidylinositol by hTAP1/SEC14L2-Mediated Lipid Exchange

**DOI:** 10.1371/journal.pone.0101550

**Published:** 2014-07-01

**Authors:** Jean-Marc Zingg, Roksan Libinaki, Mohsen Meydani, Angelo Azzi

**Affiliations:** 1 Vascular Biology Laboratory, JM USDA-Human Nutr. Res. Ctr. On Aging, Tufts University, Boston, Massachusetts, United States of America; 2 Dept. Biochem. and Mol. Biology, Monash University, Melbourne, VIC, Australia; Medical University Innsbruck, Austria

## Abstract

The vitamin E derivative, alpha-tocopheryl phosphate (αTP), is detectable in cultured cells, plasma and tissues in small amounts, suggesting the existence of enzyme(s) with α-tocopherol (αT) kinase activity. Here, we characterize the production of αTP from αT and [γ-^32^P]-ATP in primary human coronary artery smooth muscle cells (HCA-SMC) using separation by thin layer chromatography (TLC) and subsequent analysis by Ultra Performance Liquid Chromatography (UPLC). In addition to αT, although to a lower amount, also γT is phosphorylated. In THP-1 monocytes, γTP inhibits cell proliferation and reduces CD36 scavenger receptor expression more potently than αTP. Both αTP and γTP activate the promoter of the human vascular endothelial growth factor (VEGF) gene with similar potency, whereas αT and γT had no significant effect. The recombinant human tocopherol associated protein 1 (hTAP1, hSEC14L2) binds both αT and αTP and stimulates phosphorylation of αT possibly by facilitating its transport and presentation to a putative αT kinase. Recombinant hTAP1 reduces the *in vitro* activity of the phosphatidylinositol-3-kinase gamma (PI3Kγ) indicating the formation of a stalled/inactive hTAP1/PI3Kγ heterodimer. The addition of αT, βT, γT, δT or αTP differentially stimulates PI3Kγ, suggesting facilitated egress of sequestered PI from hTAP1 to the enzyme. It is suggested that the continuous competitive exchange of different lipophilic ligands in hTAPs with cell enzymes and membranes may be a way to make these lipophiles more accessible as substrates for enzymes and as components of specific membrane domains.

## Introduction

The vitamin E derivative, alpha-tocopheryl phosphate (αTP), is formed in small amounts from alpha-tocopherol (αT) in cultured cells, plasma and animal tissues and is present in foods and tissues in amounts of nmol/g of extracted material [1], [2], [3], [4]. For the phosphorylation reaction a putative αT kinase, and for the de-phosphorylation reaction an αTP phosphatase or esterase can be postulated, and both activities have been detected in cells in culture or in tissues [1], [2], [3], [5], [6].

The negative charge of αTP renders it more similar to phosphorylated messenger lipids such as phosphatidylinositol phosphates, with possibly increased ability to modulate specific and non-specific protein-membrane interactions (reviewed in [7]). However, although regulatory effects of tocopheryl phosphate esters on enzymes have been reported early on [8], the *in vivo* biological function of αTP is not clear to date. αTP may act as an active cofactor for specific enzymatic reactions (reviewed in [9]), it may be a ligand of a receptor or transcription factor, or act as “second messenger” in the membrane capable of exerting regulatory effects [2]. *In vivo*, atherosclerotic lesions of hypercholesterolemic rabbits are more efficiently reduced by supplementation with αTP when compared to α-tocopheryl acetate (αTA), resulting from reduced cytokines and scavenger receptor expression [10], [11]. The often higher potency of αTP when compared to αT is either due to a better uptake and cellular retention of the molecule e.g. by organic anion transporters (OAT) [12], to its intracellular hydrolysis by esterases [1], [2], [3], [5], [6], or to its preferential direct interaction with specific proteins and cellular structures such as with protein kinase C alpha (PKCα) [13], or similar to α-tocopheryl succinate (αTS) with Bcl-xL/Bcl-2 or mitochondrial succinate oxidase [14], [15], [16].

Since αT and to a lesser extent αTP are hydrophobic molecules located mainly in membranes, specific lipid transfer proteins (LTP) may be required to make them more accessible to kinases and phosphatases or to transport them to specific proteins, membrane domains and organelles. For the intracellular transport of αT, several proteins such as the microsomal triglyceride transfer protein (MTTP), the Niemann-Pick C1-like 1 protein, the α-tocopherol transfer protein (α-TTP) and three tocopherol associated proteins (hTAPs) (hTAP1, hTAP2, hTAP3 or hSEC14L2, hSEC14L3, hSEC14L4, respectively) have been identified (reviewed in [17]). The three hTAPs are highly homologous and related to the *Saccharomyces cerevisiae* SEC14p protein, which is the prototype of a large eukaryotic family of proteins carrying a SEC14-lipid binding domain playing a role in lipid metabolism, signalling and membrane trafficking (reviewed in [18], [19], [20], [21]). It has been postulated that these proteins stimulate signaling reactions by either directly transferring their ligands (e.g., phosphatidylinositol, phosphatidylcholine, squalene) to specific enzymes (e.g., PI3K, PI4K, phospholipase C, squalene epoxidase), by supplementing the membrane system occupied by these enzymes and regulating their activity by increasing their accessibility to further reactions [20], [22]. More recently the LTP have been suggested to sense the lipid environment and regulate enzymes by obligatory homotypic or heterotypic lipid-exchange which enables lipid presentation to the catalytic center in enzymes where they react in a temporally and spatially coordinated manner [23].

The relatively large binding pocket of hTAPs (10262 Å^3^ for hTAP1 [24]) can accommodate several different hydrophobic ligands that within cells may form a group of lipids competing for the same binding site. One group of lipids able to bind to hTAPs is related to vitamin E (α-tocopherol), encompassing the four natural tocopherol and tocotrienol analogues (α-, β-, γ-, δ-) as well as some derivatives such as α-tocopheryl quinone (αTQ) and α-tocopheryl succinate (αTS) [16], [25], [26], [27]. An intracellular tocopherol transport function of these proteins is supported by the finding that the cellular uptake of αT and αTS is increased by hTAP1 over-expression [14], [16], that the *in vitro* αT transport to mitochondria is augmented by hTAP1 [28], and that mitochondria-mediated apoptosis is induced by αTS in hTAP1-overexpressing mesothelioma cells and in prostate cancer cells [14], [15], [16].

In addition to tocopherol analogues and derivatives, hTAPs bind *in vitro* several other ligands, such as squalene, phosphatidylinositol (PI), phosphatidylinositol-3,4,5-phosphate, phosphatidylcholine (PC) and phosphatidylserine, suggesting transport of these ligands to specific enzymes or intracellular sites (reviewed in [19]). The competition with these ligands for a common binding site and their exchange could affect phospholipid-dependent transport and signalling pathways. Accordingly, αT stimulates *in vitro* squalene epoxidase and phosphatidylinositol-3-kinase gamma (PI3Kγ) activity possibly by forcing the release of squalene or phosphatidylinositol, respectively, and/or facilitating their presentation to the enzymes [14], [25], [26], [29], [30]. Thus, αT may act as competing heterotypic ligand to PI or squalene as proposed for PC in stimulating the phosphorylation of PI by PI kinases [20].

In this study, we describe the existence of αT phosphorylation activity present in primary human coronary artery smooth muscle cells (HCA-SMC). To assess the substrate specificity of the putative αT kinase, we evaluate whether other vitamin E analogues can become phosphorylated as well. Moreover, we check whether hTAP1 can bind αT and αTP and whether αT phosphorylation and PI3Kγ activity can be modulated by hTAP-mediated lipid exchange.

## Materials and Methods

### Materials

RRR-α-tocopherol (αT), RRR-β-tocopherol (βT), RRR-γ-tocopherol (γT), RRR-δ-tocopherol (δT), and RRR-α-tocopheryl quinone (αTQ) (all from Cognis, Cincinnati, OH, USA) were dissolved in ethanol as 50 mM stock solutions and the concentrations confirmed spectrophotometrically. Stock solutions (50 mM) of α-tocopheryl phosphate (αTP), γ-tocopheryl phosphate (γTP) (all provided by Phosphagenics Ltd (Melbourne, Australia)) were prepared in ethanol or water [12], [31]. Phosphatidylinositol was purchased from Sigma-Aldrich, Saint Louis, MO. Ritonavir (Moravek Biochemicals, CA, USA) was dissolved in ethanol as a 5 mg/mL stock.

### Cell culture

Primary human coronary artery smooth muscle cells (HCA-SMCs (#C-017-5C, Cascade Biologics, Portland, OR) were grown in medium 231 containing smooth muscle growth supplement (SMGS) (Cascade Biologics, Portland, OR) and 100 µg/ml streptomycin and 100 U penicillin. The human THP-1 acute monocytic leukaemia cell line (THP-1) (ATCC – TIB-202) was grown in RPMI/10% FCS, 2 mmol/L L-glutamine, 1.0 mmol/L sodium pyruvate, 4.5 g/L glucose, 100 µM of the water-soluble antioxidant L-ascorbic acid (Sigma-Aldrich, Saint Louis, MO), 100 µg/ml streptomycin and 100 U penicillin.

### Cell proliferation assay

THP-1 cells were plated into 96-well microtiter plates (10,000 cells/well), treated with αTP and γTP and grown for 0, 28 and 52 h. Treatments in 96 well microtiter plates with αTP and γTP were done using working stock dilutions prepared in 1% ethanol in order to keep total ethanol concentrations in the cell culture medium below 0.1%. Compounds diluted for the working stock dilutions were assessed by thin layer chromatography (TLC) and no loss was observed as a result of dilution (e.g. as result of precipitation). Cell numbers were assessed using the CellTiter 96 AQueous One Solution Cell Proliferation Assay (Promega, Madison, WI), and measurements were done using a GLOmax absorbance reader (Promega) at 490 nm after assay duration of 4 h.

### CD36 cell surface exposition

THP-1 cells (1×10^6^ per 10 cm dish) were treated with αTP (10 µM) or γTP (10 µM) for 24 h, harvested and CD36 cell surface exposition was analyzed by FACS as previously described using a monoclonal anti-CD36-FITC antibody (Ancell, Bayport, MN) [31], [32], [33].

### Purification of recombinant hTAP1 from *Escherichia coli*

Recombinant hTAP1 containing an amino-terminal Histidine tag was expressed and purified as previously described [26], [27].

### Binding of αTP to recombinant hTAP1

The binding of αT and αTP to recombinant hTAP1 was assessed using Isoelectric Point Mobility Shift (IPMS) assay essentially as previously described [27]. In this assay, the native hTAP1 protein migrates on an isoelectric focusing polyacrylamide gel until it has a net charge of 0 (what occurs at the calculated isolectric point of recombinant hTAP1 at pH 7.9 [27]), and the mobility of hTAP1 is changed upon ligand (PI) binding, until the PI-hTAP1 complex reaches again a net charge of 0.

### In situ tocopherol phosphorylation assay

80% confluent HCA-SMC cells in 10 cm dishes were treated with αT (50 µM) for 20 h, washed two times with PBS, washed one time with pre-warmed Intra Cellular Buffer (ICB [34], containing 120 mM KCl, 0.15 mM CaCl_2_, 10 mM EGTA, pH 7.6, 5 mM MgCl_2_), and then incubated with 2.5 ml pre-warmed ICB buffer containing 5 mM orthovanadate, 1∶25 dilution of TABS protease inhibitor cocktail (Roche), 1 mM PMSF, 1 mM DTT, 50 µg/ml digitonin (Sigma), 2 µM ATP, and 10 µM 5′-[γ-^32^P]-ATP (6000 Ci/mmol), 80 µCi/dish (Amersham Biosciences), and 20 µM α-tocopherol for 10 min. Thereafter, the cells were washed 5 times with cold PBS, and the reaction stopped with 2 ml ethanol/0.1% L-ascorbic acid. Extraction was done by adding two times 120 µl acetonitrile, vortexing 1 min, adding 2 ml hexane, vortexing 2 min, centrifuging 1 min at 3000 rpm, and the hexane phase was discarded. After that, 250 µl concentrated HCl was added to the water phase, vortexed for 1 min, and extracted two times with 2 ml hexane, vortexed 2 min and centrifuged at 3000 rpm for 2 min. The combined hexane phases were dried down under nitrogen gas and separated on Adamant TLC plates (MacheryNagel). Conditions for TLC were chloroform/methanol/water (60/40/10)(v/v/v) to 1 cm, chloroform/acetone/methanol/acetic acid/water (46/17/15/14/8)(v/v/v/v/v) to 5 cm, chloroform/n-heptan (60/40)(v/v) to 10 cm, and pure n-heptan to 13 cm.

### UPLC assay

The α-tocopheryl phosphorylation assay was performed as above, the products separated on TLC, and a control spot for αTP and two labelled sample spots scraped and extracted with ethanol. The samples were analyzed on a Waters UPLC fitted with a 1.7 µm 2.1×100 mm C18 bridged ethane linked hybrid column. The solvent chosen was A) water containing 4.0 g/L ammonium bicarbonate, and B) methanol. The flow rate was at 0.4 ml/min and column temperature was 40°C. The following gradient was used (Table 1).

**Table 1:** Conditions for detection of αTP by UPLC.

Time (min)	Flow Rate (ml/min)	%A	%B	Curve
1. Initial	0.400	15.0	85.0	Initial
2. 4.00	0.400	3.0	97.0	6
3. 5.00	0.400	15.0	85.0	11

### Phosphatidylinositol kinase assay

The *in vitro* phosphatidylinositol kinase assay was performed using recombinant PI3Kγ/p110γ basically according to the protocol supplied by the manufacturer (Alexis Biochemicals, San Diego, CA). Briefly, sonicated phosphatidylinositol (100 µM), tocopherols (50 µM) and the recombinant hTAP1 protein (100 nM) were preincubated for 10 min in a total volume of 100 µl reaction buffer (20 mM Tris-HCl (pH = 7.4), 4 mM MgCl_2_, 100 mM NaCl) containing 20 µM cold ATP and 10 µCi -[γ-^32^P]-ATP (Amersham Biosciences). The reaction was started by adding 0.2 µg PI3Kγ/p110γ (50 nM) and incubated at 37°C for 20 min. The reaction was stopped with 150 µl of 1 M HCl, and the phospholipids extracted with 400 µl chloroform/methanol (1∶1), separated by TLC, exposed to film and quantified as previously described [26].

### Transfection

THP-1 cells (1.5×10^6^ cells per ml) were grown in 12 well plates (1 ml per well) overnight, transfected with pCGCG-luc (a reporter plasmid containing 3169 bp of the human VEGF promoter in front of the *Firefly* luciferase gene (kindly provided by S. J. Prior, University of Maryland, Baltimore, MD [35])), and with the *Renilla* internal control plasmid pRL-TK (Promega, Madison, WI), for 3 h using Fugene (Promega) as transfection reagent, and then treated with αT, γT, αTP or γTP (all 20 µM) for additional 21 h. Extracts were prepared, and promoter activities were measured using the Dual-Luciferase assay kit (Promega) using a GLOmax luminometer (Promega). The VEGF promoter-*Firefly* luciferase activities were normalized to the thymidine kinase promoter-*Renilla* luciferase activities, and the activities of the control transfections were set to 100%.

### Western blotting

THP-1 monocytes (1.5×10^6^ cells in 10 ml media per dish) were grown overnight and then treated with αT, αTP and AS-605240 for 24 h as indicated in the figure legend. The cells were harvested, centrifuged, washed with ice cold PBS, incubated at 4°C for 5 min in 0.5 mL cell lysis buffer (20 mM Tris pH 7.5, 150 mM NaCl, 1 mM EDTA, 1 mM EGTA, 1% Triton X-100, 2.5 mM sodium pyrophosphate, 1 mM β-glycerol phosphate, 1 mM sodium orthovanadate, 1 mM PMSF, 1/1000 diluted TABS protease inhibitor cocktail (Roche, Indianapolis, IN)), homogenized 10 times using a G26 needle and centrifuged for 10 min at 16000 rcf at 4°C. The protein concentration was measured using the BCA kit (Pierce, Rockford, IL). Immunoblots were done according to standard methods using 30 µg of extract per lane and separated by 10% SDS-PAGE. The level of Akt phosphorylation was determined using primary anti-phospho-Akt(Ser473) antibody, primary anti-Akt antibody (both from Cell Signalling Technology, Danvers, MA), and horseradish peroxidase coupled donkey anti-rabbit IgG secondary antibody (Amersham Biosciences, Piscataway, NJ). Proteins were visualized with an enzyme-linked chemiluminescence detection kit (Immun-Star HRP) according to the manufacturer's instructions (Biorad, Hercules, CA). Chemiluminescence was monitored by exposure to film (Kodak BioMax), and the signals were analyzed using a Fluorchem 8900 workstation and the AlphaEaseFC software (AlphaInotech).

### Statistical analysis

All values are expressed as the mean ± standard error of the mean (SEM) as explained in the figure legends. The median fluorescence intensity was determined for FACS analysis and the mean ± SEM calculated as described in the figure legends. Student's *t*-test was used to analyze the significant differences between two conditions. A *p*<0.05 was considered as significant and indicated by * or # in the graphs.

## Results

### Phosphorylation of αT in primary human coronary artery smooth muscle cells

**Figure 1:**
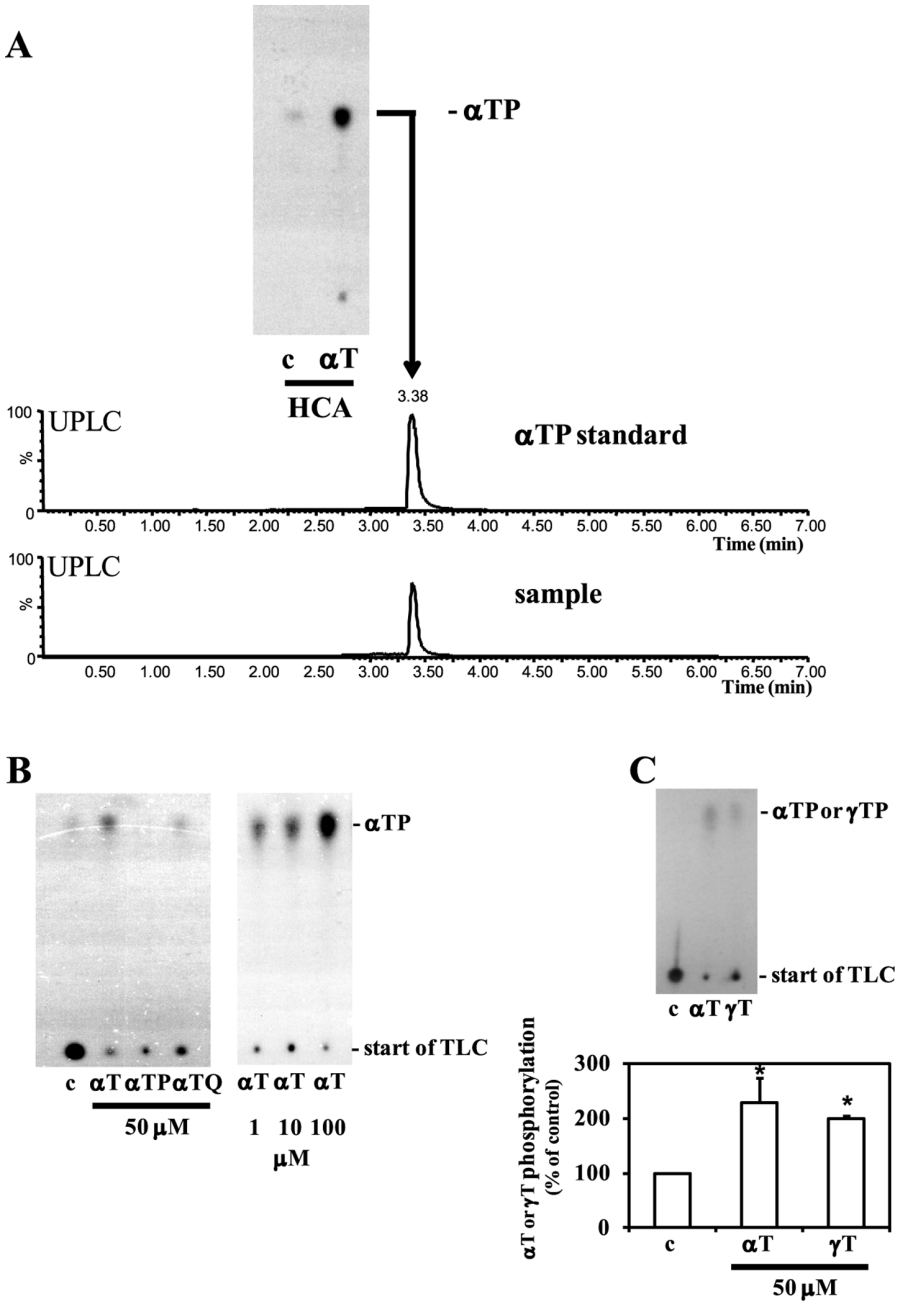
Primary human coronary artery smooth muscle cells (HCA-SMC) contain αT phosphorylation activity. (A) HCA-SMC cells were treated with 0.1% ethanol control (c) or αT, and the *in situ* phosphorylation reaction, lipid extraction, and thin layer chromatography performed as indicated in materials and methods. The TLC plate was subsequently exposed to film, and the labeled sample spots and control spots separated in parallel were scraped, extracted and the presence of αTP confirmed by UPLC (lower part). (B) Specificity of the αT phosphorylation reaction, concentration dependency and substrate specificity of αT phosphorylation. HCA-SMC cells were treated with 0.1% ethanol control (c) or αT, αTP, αTQ at the indicated concentrations, and the phosphorylation reaction, lipid extraction, and thin layer chromatography (TLC) were performed as indicated in materials and methods. (C) Comparison of αT and γT phosphorylation (mean±SEM, n = 2, *P<0.05 relative to control (c)).

In preliminary studies we have shown that small amounts of αT can become phosphorylated *in vitro* by HMC-1 human mast cells and primary human coronary artery cells [2], as well as in NIH-3T3-L1 adipocytes and in rat livers upon feeding ^14^C-αT [3]. To characterize the enzymatic reaction involved, an *in situ* αT phosphorylation assay measuring the production of αTP from αT and [γ-^32^P]-ATP was performed with primary human coronary artery smooth muscle cells (HCA-SMC), the newly formed αTP extracted and separated by Thin Layer Chromatography (TLC) (Figure 1A). After that, the labelled spot corresponding to αTP was scraped from the TLC plate and analyzed by Ultra Performance Liquid Chromatography (UPLC) as described in Materials and Methods. In the UPLC graph, the peaks from the isolated spots corresponded with the αTP control peaks, clearly showing that αTP is synthesized in our *in vitro* assay system (Figure 1A). A very weak spot was observed in the absence of added αT, reflecting some αT in the serum. The phosphorylation of αT occurred in a concentration dependent manner. No phosphorylation occurred with αTP suggesting that the pyrophosphate is not formed. The phosphorylation of α-tocopheryl quinone (αTQ) and γT was measured as well, although with lower efficiency (Figure 1B and C). By comparing the intensity of the radioactive αTP spots with spots obtained from diluting 5′-[γ-^32^P]-ATP (6000 Ci/mmol) of known concentration, it was calculated that ∼168 molecules/cell/hour were synthesized in this assay.

### Comparison of cellular activities of αTP and γTP

**Figure 2:**
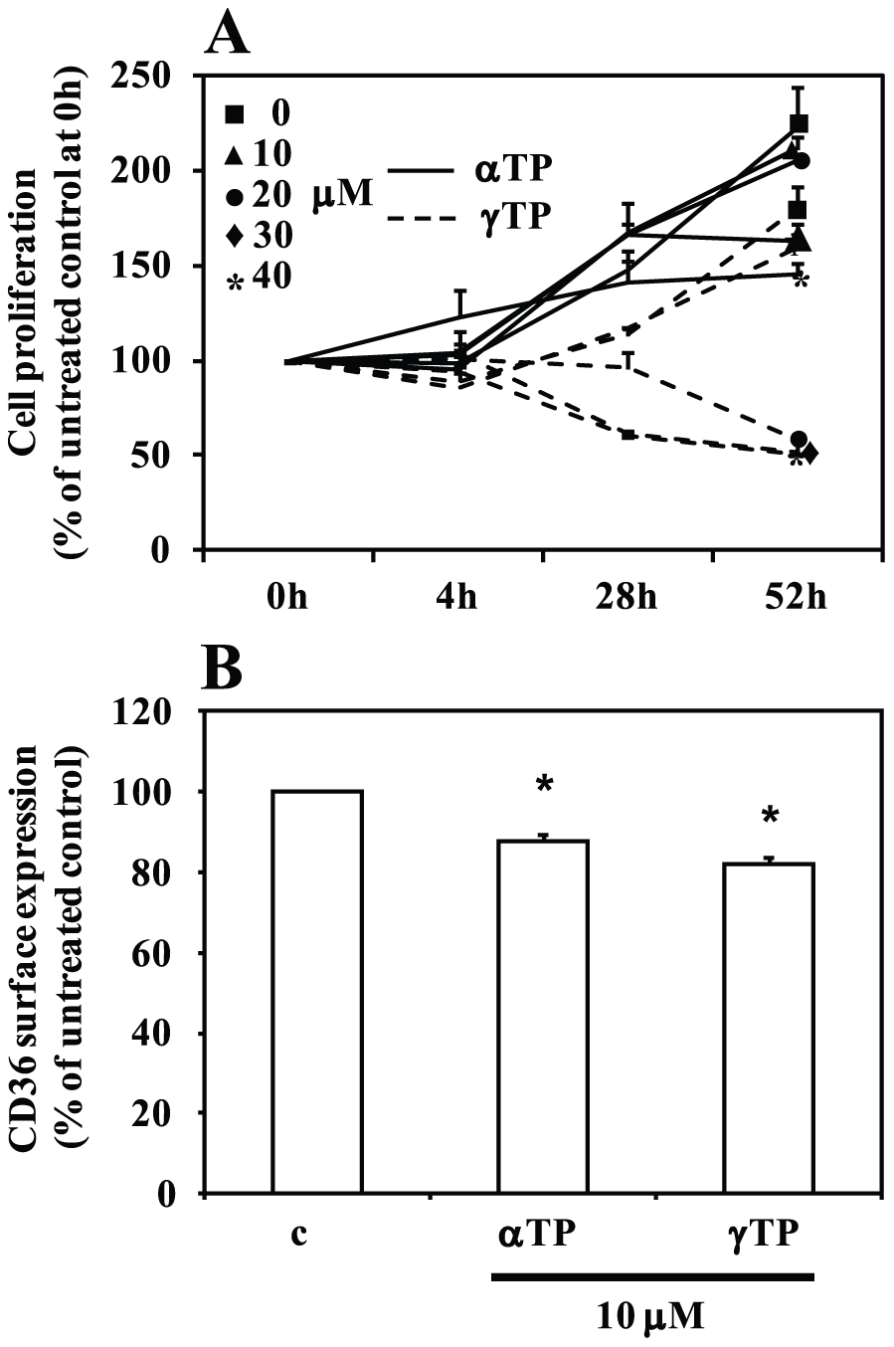
Comparison of cellular activities of αTP and γTP. (A) Inhibition of THP-1 cell proliferation by αTP or γTP (both at 0, 10, 20, 30, 40 µM) after 4 h, 28 h and 52 h treatment (mean±SEM, n = 4, relative to untreated control at 0 h set to 100%). (B) Inhibition of CD36 cell surface exposition as analyzed by FACS after treatment with αTP (10 µM) or γTP (10 µM) for 24 h (mean±SEM, n = 4, *P<0.05 relative to control (c)).

Since both αTP and γTP are formed in the *in vitro* assay, it was interesting to determine whether the two compounds affect cells with different potency, what could contribute to the activity differences seen with αT and γT in THP-1 monocytic leukaemia cells [4], [36] and other experimental systems despite a generally lower γT level (reviewed in [37]). When THP-1 cells were incubated with either αTP or γTP at increasing concentrations for 4, 28 or 52 h, γTP inhibited their proliferation more efficiently than αTP (Figure 2A); concentrations of γTP above 20 µM led to cell loss due to cytotoxic/apoptotic effects, what occurred with αTP only at concentrations above 46 µM [31]. Similar to that, γTP inhibited CD36 scavenger receptor surface exposition stronger than αTP (Figure 2B). It remains to be shown whether the higher activity of γTP contributes to the higher activity of γT when compared to αT observed in a number of experimental models such as apoptosis, cell proliferation, gene expression, cancer and inflammation (reviewed in [38]).

### Binding of α-tocopherol and α-tocopherol phosphate to human tocopherol associated protein 1 (hTAP1) is associated with release of bound phosphatidylinositol

**Figure 3:**
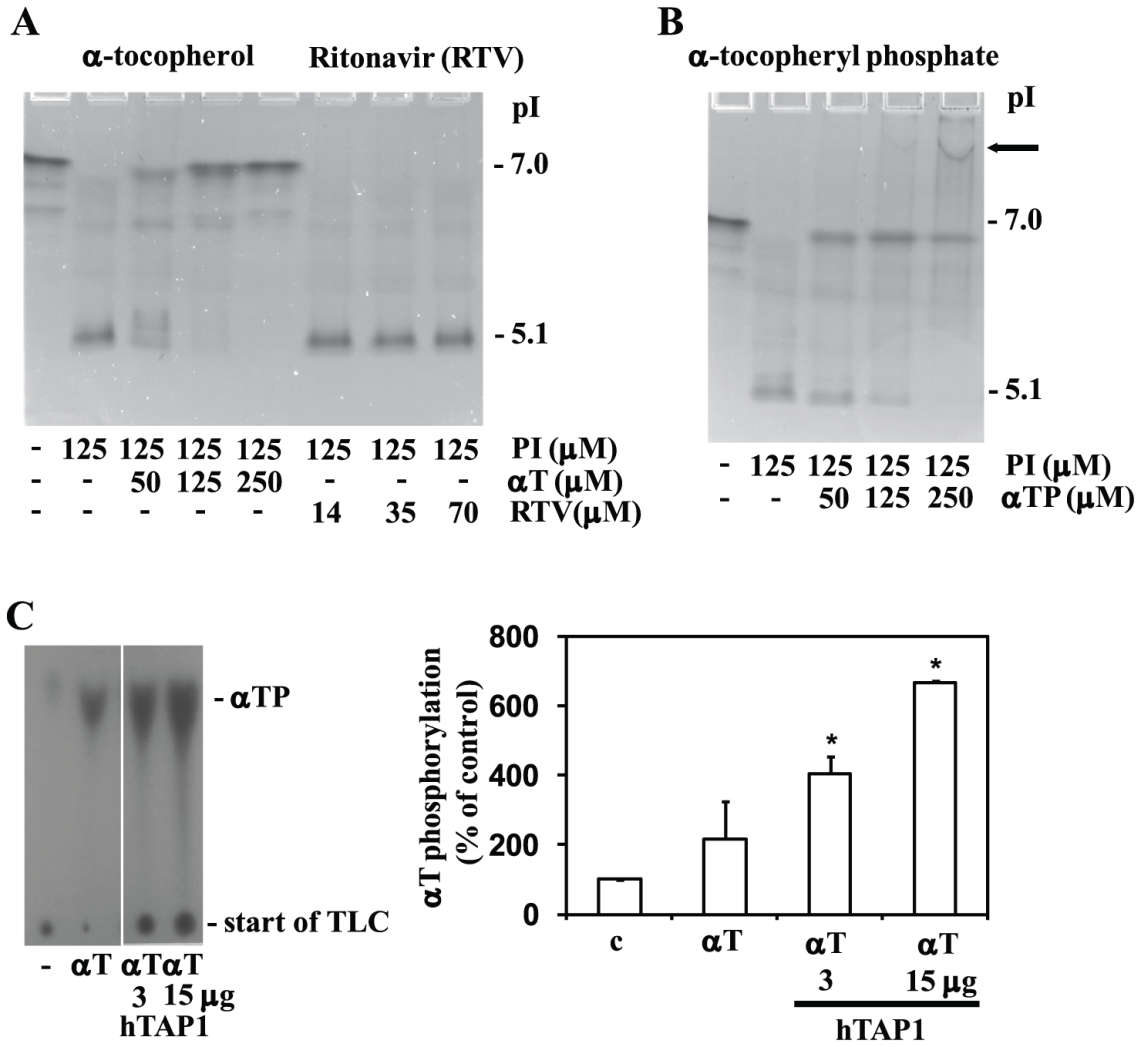
Binding of αT and αTP to recombinant hTAP1 and lipid exchange. (A) Isoelectric Point Mobility Shift assay (IPMS) shows competition of αT but not of ritonavir (negative control) with phosphatidylinositol (PI). Recombinant hTAP1 (30 µg) was incubated with αT, ritonavir, and PI at the indicated concentrations and the IPMS assay performed as described in Materials and Methods. Gels were stained using a mixture of Coomassie Blue and Crocein Scarlet. (B) Isoelectric mobility shift assay shows competition of αTP with PI. The arrow indicates a supershift probably resulting from detergent-like denaturing effects of αTP at high concentrations. The experiments have been repeated twice with similar results. (C) Stimulation of αT phosphorylation reaction with recombinant hTAP1. HCA-SMC cells were treated with 0.1% ethanol control (c) or αT (50 µM) and two different amounts of recombinant hTAP1 (3 and 15 µg/2.5 ml ICB). The phosphorylation reaction, lipid extraction, and thin layer chromatography were performed as indicated in materials and methods, the control set to 100% and the mean±SEM of two experiments plotted (*P<0.05 relative to control (c)).

**Figure 4:**
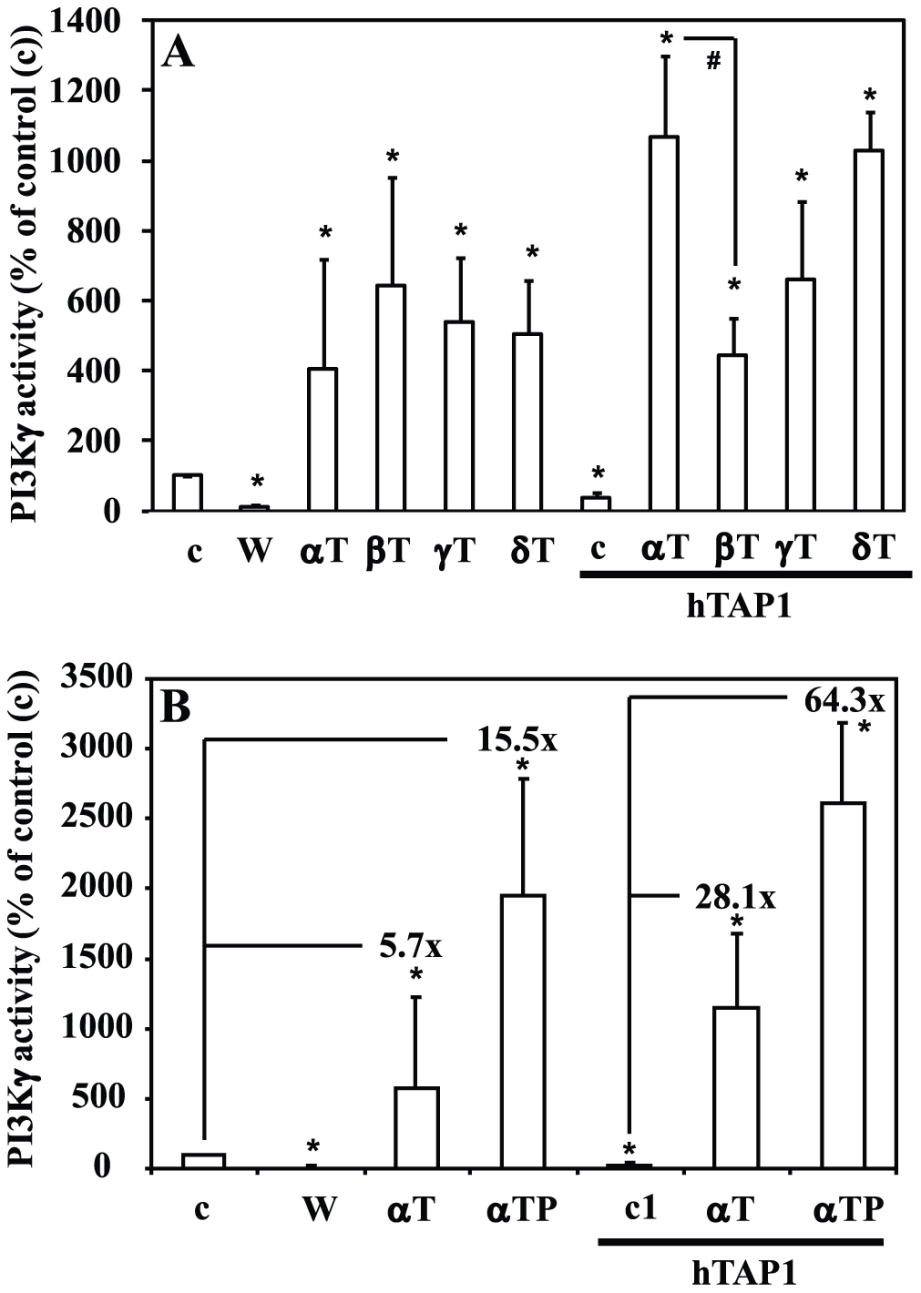
Stimulation of phosphatidylinositol-3-phosphate kinase gamma (PI3Kγ) activity with different tocopherol analogues. (A) *In vitro* PI3Kγ activity is modulated by recombinant hTAP1 (4 µg) in a tocopherol analogue specific manner. PI3Kγ activity was assessed as described in materials and methods and the mean±SEM results plotted (n = 3, *P<0.05 relative to untreated control (c) without hTAP1; ^#^ *P*<0.05 relative to αT in the presence of hTAP1). αT, βT, γT, δT: α-, β-, γ-, δ-tocopherols, respectively. W: wortmannin. (B) *In vitro* PI3Kγ activity is inhibited by wortmannin (W) (1 µM), and stimulated by αT (50 µM) and more by αTP (50 µM). Recombinant hTAP1 (4 µg) inhibits PI3Kγ activity possibly by forming a stalled/inactive complex; addition of αT or αTP reverts the inhibition by hTAP1, possibly by promoting dissociation of the inactive complex and/or competing with bound phosphatidylinositol allowing its egress from the hTAP1 binding site and the transfer to the enzyme. PI3Kγ activity was assessed as described in materials and methods, the control set to 100% and the mean±SEM plotted (n = 3, *P<0.05 relative to control (c)).

hTAP1 can bind several uncharged hydrophobic ligands (such as tocopherols, tocotrienols, phosphatidylcholine, phosphatidylserine and squalene), but also charged ligands (such as αTS, phosphatidylinositol (PI) and phosphatidylinositol-3,4,5-phosphate) (reviewed in [19]). It was therefore important to check whether it can also bind αTP, which with calculated pK_a_ values of 6.07 and 1.64, is expected to carry two negative charges with physiological condition and occurs in solution as di-sodium salt [39]. Indeed, when assayed *in vitro* by Isoelectric Point Mobility Shift (IPMS) assay [27], αT could compete with PI for binding to recombinant hTAP1 suggesting that the two ligands bind and depending on their concentration can exchange each other at an overlapping binding site (Figure 3A). Since 50% displacement of PI (125 µM) was observed with αT at 50 µM, the affinity of αT to the binding pocket of hTAP1 is stronger than that of PI. When compared to αT (Figure 4A), the competition with αTP was slightly weaker (Figure 3B). As negative control, another hydrophobic molecule, ritonavir, was not able to compete, showing the specificity of this assay (Figure 3A).

### The α-tocopheryl phosphorylation reaction is stimulated by recombinant hTAP1

Having established that αT and αTP both can bind to hTAP1, it was important to assess whether hTAP1 facilitates the phosphorylation reaction of αT. Indeed, the addition of recombinant hTAP1 (3 and 15 µg/2.5 ml ICB) stimulated the αT phosphorylation reaction in a concentration dependent manner (Figure 3C).

### The phosphatidylinositol-3-kinase gamma activity is stimulated *in vitro* by αT and αTP in an hTAP1-dependent manner

In a previous study, recombinant hTAP1 reduced the *in vitro* activity of the phosphatidylinositol-3-kinase gamma (PI3Kγ); the addition of αT stimulated PI3Kγ, e.g. by forcing egress of PI from hTAP1 to the enzyme and/or by inducing conformational changes leading to activation of PI3Kγ [14], [26]. To assess whether different tocopherol analogues influence PI3Kγ activity with different potency, we measured *in vitro* the activity of recombinant PI3Kγ in the presence of αT, βT, γT and δT. All four tocopherols stimulated PI3Kγ activity with similar efficiency (Figure 4A).

In the presence of recombinant hTAP1, *in vitro* PI3Kγ activity was reduced (to 38±24%, n = 3, *P*<0.05) what could be the result of direct hTAP1/PI3Kγ interaction and/or formation of an inactive/stalled complex (Figure 4A) [26]. In the presence of hTAP1 the different tocopherol analogues showed different potency to stimulate PI3Kγ (Figure 4A), suggesting that hTAP1 not only reduces PI3Kγ activity, but also gives a certain selectivity to the tocopherols to activate PI phosphorylation by PI3Kγ, e.g. as a result of different binding affinity, ligand exchange rate or ligand induced conformational changes.

Since αTP can also bind hTAP1 (Figure 3B), it was important to determine whether hTAP1 can influence the ability of αTP to stimulate PI3Kγ activity. αTP stimulated PI3Kγ stronger than αT (Figure 4B). In the presence of hTAP1 the fold induction of PI3Kγ activity seen with αTP was even higher, despite having a slightly lower ability to compete with PI (Figure 3A and 3B), what may play a role in the observed enhanced activation of the PI3K/Akt pathway by this compound [4].

### αT, αTP, γT, and γTP differentially up-regulate vascular endothelial growth factor promoter activity in THP-1 cells

**Figure 5:**
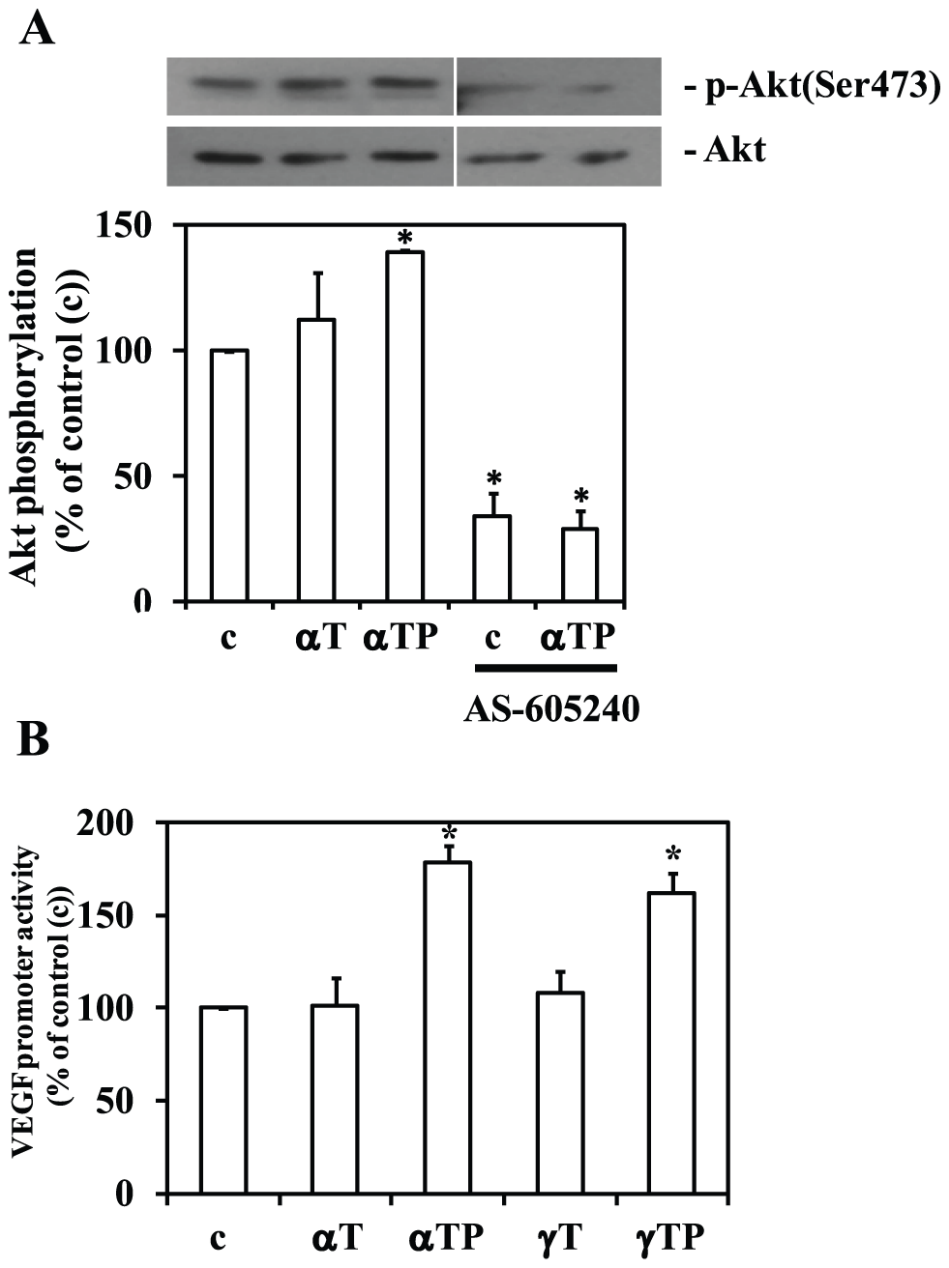
PI3Kγ is involved in stimulating Akt(Ser473) phosphorylation by αTP in THP-1 monocytes. (A) THP-1 monocytes were incubated with or without αT or αTP (both 40 µM) or the specific PI3Kγ inhibitor AS-605240 (1 µM) for 24 h and western blots performed as described in materials and methods (n = 3, *P<0.05 relative to untreated control (c)). (B) Differential regulation of VEGF promoter activity by tocopherol analogues (all 20 µM) in THP-1 monocytes. αTP and γTP significantly induce the VEGF promoter activity in THP-1 monocytes, whereas αT and γT had no effect (n = 4, *P<0.05 relative to untreated control (c)).

We previously reported that αTP stimulates the PI3K/Akt signal transduction pathway, leading to the induction of a number of genes including the vascular endothelial growth factor (VEGF) [4]. To assess whether PI3Kγ is the PI3K isoform regulating VEGF expression in THP-1 monocytes, these cells were treated with AS-605240 (1 µM), an inhibitor specific for PI3Kγ [40], [41]. As measured by Western blotting, Akt(Ser473) phosphorylation was strongly inhibited by AS-605240 (to 34.1±15.7, n = 3, p<0.05) and the stimulation by αTP was blocked (Figure 5A), suggesting that PI3Kγ is a predominant PI3K isoform present in these cells [40], and therefore is involved in regulating Akt and VEGF by αTP [4]. To assess whether αT, αTP, γT, and γTP differentially up-regulate VEGF promoter activity in THP-1 cells, a reporter construct containing the human VEGF promoter in front of the luciferase gene was transfected into THP-1 monocytes and VEGF promoter activity measured. Both αTP and γTP significantly activated the VEGF promoter with similar potency, whereas αT and γT had no significant effect in these cells (Figure 5B).

## Discussion

We show here that αT and γT is phosphorylated in HCA-SMC suggesting presence of enzyme(s) with αT phosphorylation activity in these cells. The αT phosphorylation activity is stimulated by recombinant hTAP1 which binds αT and αTP thus facilitating the transport and presentation of the substrate (αT) to the putative αT kinase and/or the removal of the product (αTP) away from it, thus increasing the enzyme's catalytic turnover. The *in vitro* activity of PI3Kγ is inhibited by hTAP1 indicating the formation of an inactive hTAP1/PI3Kγ heterodimer [26]. The binding of PI to hTAP1 is reversed by αT and αTP leading to stimulation of PI3Kγ activity, suggesting that αT and αTP promote dissociation of the inactive complex and/or the release of sequestered PI from hTAP1 for subsequent presentation to the kinase by means of a heterotypic lipid exchange mechanism. Although it is possible that presentation of αT to the putative αT kinase occurs by homotypic exchange, it should be noted that αT phosphorylation activity was assayed in the presence of permeabilized cells possibly allowing heterotypic ligand exchange when encountering the cellular plasma membranes [20]. Analogous lipid-exchange mechanisms were recently visualized with the crystal structures of the closest SEC14p homolog - the *Saccharomyces cerevisiae* Sfh1a [42], as well as proposed for human α-TTP [43], [44].

While our *in vitro* and cell culture studies are focusing only on PI3Kγ and αT kinase, other enzymes such as PI4K, phospholipases, squalene epoxidase, fatty acid synthase, choline-phosphate cytidyltransferases could be modulated by hTAPs-mediated lipid exchange as well [30]. At a molecular level, the transfer of PI/PC by *Saccharomyces cerevisiae* SEC14p function has been mainly linked with activation of PI4K, secretion and trafficking of lipid raft proteins [42], [45]. However, none of the three hTAP1/2/3 proteins was able to complement for SEC14p function in yeast [26], and direct interaction of the hTAPs with PI3K and modulation of its activity *in vitro* and *in vivo* in mice and humans suggests that these proteins are performing a regulatory function different from yeast SEC14p [14], [26]. hTAPs may affect gene expression in a tocopherol- and/or tocopheryl phosphate- dependent manner, e.g. by affecting the PI3K/Akt signal transduction pathway by transporting these ligands to specific enzymes such as cytosolic PI3Kγ, or to membrane sites accessible for regulating PI3K, Akt, and PHLPP1 [4], [46], [47], [48]. Whether similar signalling events also contribute to the regulation of the biosynthesis of cholesterol by TAP1/SEC14L2 by regulating squalene epoxidation via stimulating squalene transport and presentation to squalene epoxidase remains to be investigated [29], [30].

**Figure 6:**
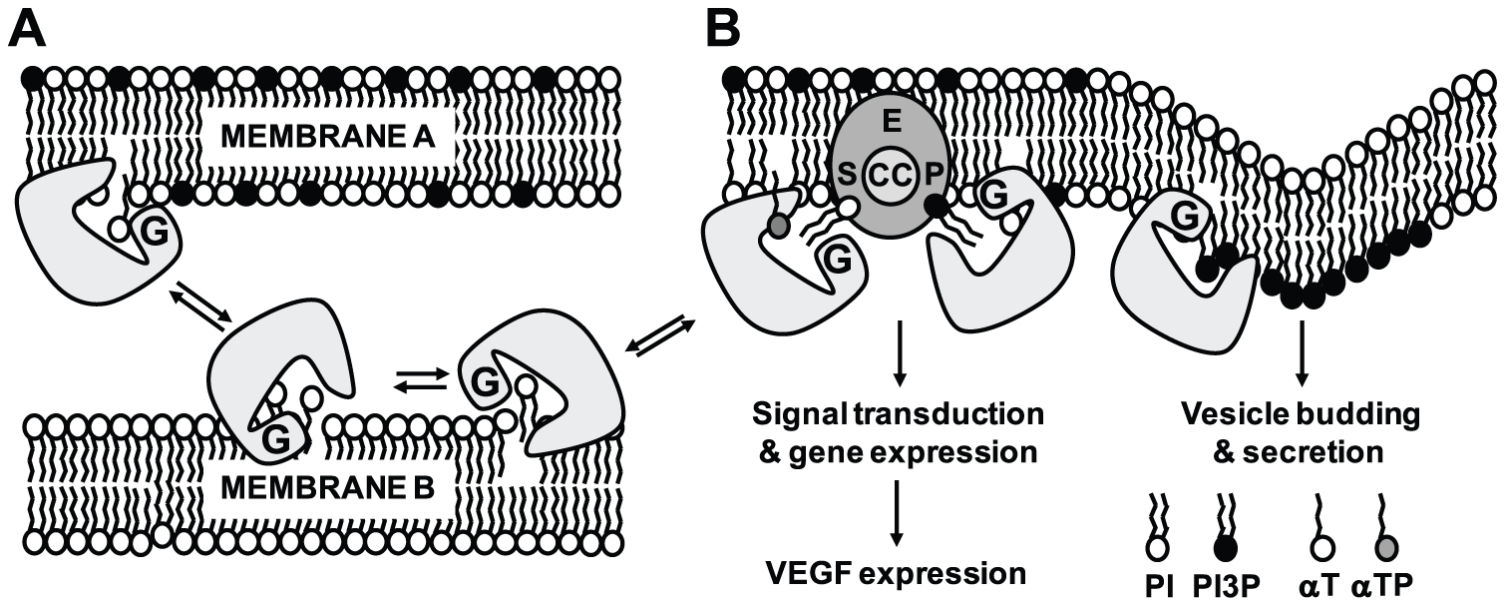
Hypothetical molecular model for hTAPs in lipid transport and enzyme regulation. (A) hTAPs transfer lipids from/to cellular import/export sites or between different membranes and membrane domains such as lipid rafts, e.g. between membranes of the Golgi, endoplasmic reticulum, mitochondria, vesicles or membranes of cilia in airway epithelia [28], [51]; in secretory cells lipid transfer may be polarized. (A and B) hTAPs mediated lipid transport may change lipid composition and membrane curvature and in this way influence signal transduction and secretion. (B) hTAPs bring lipid substrates (S) to specific enzymes (E), present them in the correct orientation and timing, and/or remove the lipid products (P) from the enzyme, thus enhancing lipid turnover at the catalytic center (CC). Lipid exchange may occur preferentially upon interaction of hTAPs with membranes, thus confining lipid presentation by hTAPs and subsequent lipid modification to enzymes located to membranes. Moreover, the affinity of different ligands to the ligand binding pocket can influence lipid exchange rate thus influence lipid-specificity to stimulate enzyme activity. The carboxy-terminal GOLD (G) domain in hTAPs may confine the exchange activity to certain sites and thus further increase the reaction specificity.

The exchange of hTAP ligands may be a way to make these lipophiles more accessible as substrates for enzymes and as components of specific membrane domains (lipid rafts, vesicles, organelles) (Figure 6A and 6B). Each hTAP may show different preferences for specific lipids and enzymes what determines which lipids are exchanged and which reaction is catalysed. It has to be kept in mind that the activity measured in our assay represents the sum of many binary on/off switches at individual hTAP1/PI3Kγ molecules and their response to αT and αTP. In cells, in which hTAP1/PI3Kγ interaction occur dynamically in time and space, hTAP1 may act as sensor for lipid information (location, type and amount of lipid, lipid gradients) and generate a self-organizing system able to respond to changes in extra- and intracellular lipids and transmit this information into responses of PI3K-mediated signalling and gene expression. In fact, the higher concentration of vitamin E in plasma membrane domains (e.g. lipid rafts) is compatible with specific mechanisms for tocopherol insertion and removal from membranes, vesicles and organelles [49], [50] and may define the sites at which αT or αTP mediated lipid exchange and signaling can occur. Enhanced tocopherol delivery to membranes may be required in cells at risk for tocopherol depletion, such as in epithelial duct cells of secretory glands or airway ciliated epithelial cells exposed to high levels of oxygen, in which the hTAPs are abundantly expressed [28], [51]. Enrichment of αT, αTP, or PI3P/PI4P at specific sites may also contribute to the αT-mediated increase of hexosaminidase secretion in rat mast cells [52], and/or determine the identity of vesicles required for intracellular trafficking [53]. It is noteworthy that the related *Saccharomyes cerevisiae* SEC14p mediates vesicle formation and secretion from endosomes and the trans-Golgi Network (TGN) to the vacuole [54], and it can be speculated that the lumen of the epithelial ducts represents a cellular space that is homologous to the yeast vacuole.

As described in this study, αTP facilitates better lipid exchange in hTAPs when compared to αT, thus stimulating better hTAPs-dependent reactions by enhancing the egress and presentation of heterotypic ligands to enzymes in the correct spatial orientation. The cellular response to αT and αTP may depend on whether they enable catalysis of PI by PI3K or PI4K, or the conversion of αT to αTP by a putative αT kinase or vice versa by an αTP phosphatase in a given tissue and cell type [4]. We find that αTP (and more so γTP) is more potent than αT in reducing cell proliferation, and in normalizing oxLDL-induced CD36 mRNA and protein expression [31] as well as CD36 cell surface exposition [4]. Since CD36 mediates signal transduction and gene expression of ligands that increase VEGF expression (e.g. oxLDL and oxidized lipids [55], [56], [57]), these changes in CD36 expression and localization may further contribute to the regulatory effects of αT or αTP on VEGF expression [58], [59]. Interestingly, one ligand of CD36, thrombospondin, negatively regulates VEGF expression and angiogenesis [60] as well as myristic acid uptake and signalling [61], and it remains to be determined whether αT or αTP uptake and interaction with and internalization of CD36, and possibly interference with thrombospondin binding, play some role in stimulating VEGF expression.

The production of αTP in vascular smooth muscle cells (VSMC) may instruct neighbouring pericytes/endothelial cells or invading monocytes/macrophages to produce VEGF leading to an increase of vascular permeability and/or adaptive formation of new vessels [62], [63], e.g. during post-infarction wound healing [64] or during development acting as tubulogenic morphogen during vasculo- and/or nephro-genesis [65]. Whether activation of PI3K/Akt/VEGF and angiogenesis/vasculogenesis by αTP mediates the essential function of αT to prevent fetal resorption and ischemia/reperfusion injury in placenta, embryo, brain and muscle remains to be further investigated [66]. It appears possible that usage of αT/αTP-induced PI3Kγ-mediated signalling to enhance angiogenesis e.g. during placentation and embryogenesis has evolved to ensure sufficient amounts of αT to prevent free radicals damage upon formation of functional oxygen-transporting blood vessels. In the mammary gland epithelium, an increase in VEGF expression by αTP could also assist in the expansion of the vascular and lobulo-alveolar system during pregnancy and lactation, and increase capillary permeabilization required to increase the production of milk with sufficient VEGF and αT to nurse pups [67], [68]. It is interesting to note that in the mammary gland TAP proteins are expressed specifically in epithelial duct cells where they may take part in regulating these events [28].

In summary, the activities described with hTAP1/αT-kinase and hTAP1/PI3Kγ fit well into a model proposed for *Saccharomyces cerevisiae* SEC14p-related proteins [20], [23], [69], [70]. However, whether these reactions play a role for *in vivo* signalling function of αT and αTP requires the cloning of an αT kinase as well as an αTP phosphatase. Ligand exchange for sites within hTAPs could be a way to enhance PI3Kγ (or other enzymes) -dependent lipid reactions and increase their specificity in time and space. In doing so, hTAP/PI3K can act as sensor for cellular lipid information (location, type and amount of lipid) and translate it into responses in signalling and gene expression. It can also be envisioned that hTAPs catalyze lipid reactions not only at nano-scale for lipid transfer and signalling in cells, but also at larger scale for applications in biotechnology. Further research is required to identify the putative αT kinase, to establish the biological function of αTP and γTP and the role of the three hTAPs in lipid transport, signal transduction and gene expression.
